# Foot-and-mouth disease virus VP1 degrades YTHDF2 through autophagy to regulate IRF3 activity for viral replication

**DOI:** 10.1080/15548627.2024.2330105

**Published:** 2024-03-22

**Authors:** Huisheng Liu, Qiao Xue, Fan Yang, Weijun Cao, Pengfei Liu, Xiangtao Liu, Zixiang Zhu, Haixue Zheng

**Affiliations:** State Key Laboratory for Animal Disease Control and Prevention, College of Veterinary Medicine, Lanzhou University, Lanzhou Veterinary Research Institute, Chinese Academy of Agricultural Sciences, Lanzhou, China

**Keywords:** Autophagy, FMDV, IRF3, GTPBP4, YTHDF2, MTOR

## Abstract

Many viruses, including foot-and-mouth disease virus (FMDV), can promote the degradation of host proteins through macroautophagy/autophagy, thereby promoting viral replication. However, the regulatory mechanism between autophagy and innate immune responses is not fully understood during FMDV infection. Here, we found that the host GTPBP4/NOG1 (GTP binding protein 4) is a negative regulator of innate immune responses. GTPBP4 deficiency promotes the antiviral innate immune response, resulting in the ability of GTPBP4 to promote FMDV replication. Meanwhile, GTPBP4-deficient mice are more resistant to FMDV infection. To antagonize the host’s antiviral immunity, FMDV structural protein VP1 promotes the expression of GTPBP4, and the 209th site of VP1 is responsible for this effect. Mechanically, FMDV VP1 promotes autophagy during virus infection and interacts with and degrades YTHDF2 (YTH N6-methyladenosine RNA binding protein F2) in an AKT-MTOR-dependent autophagy pathway, resulting in an increase in *GTPBP4* mRNA and protein levels. Increased GTPBP4 inhibits IRF3 binding to the *Ifnb/Ifn-β* promoter, suppressing FMDV-induced type I interferon production. In conclusion, our study revealed an underlying mechanism of how VP1 negatively regulates innate immunity through the autophagy pathway, which would contribute to understanding the negative regulation of host innate immune responses and the function of GTPBP4 and YTHDF2 during FMDV infection.

**Abbreviation:** 3-MA:3-methyladenine; ACTB: actin beta; ATG: autophagy related; ChIP:chromatin immunoprecipitation; CQ: chloroquine; DAPI:4’,6-diamidino-2-phenylindole; dpi: days post-infection; EV71:enterovirus 71; FMDV: foot-and-mouth disease virus; GTPBP4/NOG1: GTPbinding protein 4; HIF1A: hypoxia inducible factor 1 subunit alpha;hpt:hours post-transfection; IFNB/IFN-β:interferon beta; IRF3: interferon regulatory factor 3; MAP1LC3/LC3:microtubule associated protein 1 light chain 3; MAVS: mitochondriaantiviral signaling protein; MOI: multiplicity of infection; MTOR:mechanistic target of rapamycin kinase; m6A: N(6)-methyladenosine;qPCR:quantitativePCR; SIRT3:sirtuin 3; SQSTM1/p62: sequestosome 1; STING1: stimulator ofinterferon response cGAMP interactor 1; siRNA: small interfering RNA;TBK1: TANK binding kinase 1; TCID_50_:50% tissue culture infectious doses; ULK1: unc-51 like autophagyactivating kinase 1; UTR: untranslated region; WT: wild type; YTHDF2:YTH N6-methyladenosine RNA binding protein F2

## Introduction

Foot-and-mouth disease virus (FMDV) is a single-stranded positive-sense RNA virus that causes foot-and-mouth disease (FMD) in domestic and wild cloven-hoofed animals worldwide [1,2]. There are seven known serotypes of FMDV (A, O, Asia1, C, SAT1, SAT2, and SAT3) and multiple subtypes. FMDV contains a genome of approximately 8.5 Kb, which encodes a single polyprotein that is post-translationally cleaved into mature structural and non-structural proteins including L^pro^, VP1, VP2, VP3, VP4, 2A, 2B, 2C, 3A, 3B, 3C^pro^, and 3D^pol^ [3]. FMDV VP1 is a major structural protein and contains neutralizing antigenic sites and the highly conserved arginine-glycine-aspartate/RGD residues. VP1 plays an important role in virus adhesion, invasion, and immune protection serotypes. Therefore, the nucleotides of VP1 have been used to determine the serotypes of picornaviruses [4,5]. In addition, VP1 could induce apoptosis via the Akt signaling pathway and suppress the type I interferon [6]. For instance, FMDV VP1 targets the adaptor molecule MAVS (mitochondrial antiviral signaling protein) to inhibit type I interferon signaling [7], and VP1 antagonizes MAP3K8/tumor progression locus 2-mediated activation of the IRF3 (interferon regulatory factor 3) signaling pathway to facilitate viral replication [8].

To counteract host antiviral responses and maintain viral replication, the virus must overcome host innate immune responses to establish an effective infection. After RNA virus infection, RIGI recruits MAVS to activate TBK1 (TANK binding kinase 1), resulting in the activation of IRF3 and IRF7, which induces the production of type I interferon (IFNA and IFNB) [9]. IRF3 is a common molecule in all innate immune signaling pathways [10]. Upon viral infection, cytoplasmic IRF3 is phosphorylated and forms dimers. Subsequently, IRF3 enters the nucleus and associates with CREBBP (CREB binding protein)-EP300/p300 coactivators to form a complex and binds to the promoter of the targeted genes, leading to the transcription of interferon and the downstream ISG and IFIT targets [11,12]. Some negative regulators have been identified to inhibit IRF3 functions. For example, the cell growth-regulating nucleolar protein LYAR suppresses IFN production by targeting phosphorylated IRF3 [13], OTUD7B deubiquitinates SQSTM1/p62 and promotes IRF3 degradation to inhibit antiviral immunity [14], and the prolyl isomerase PIN1 negatively regulates the innate antiviral response by proteasome-dependent degradation of IRF3 [15]. The negative regulation of IRF3-mediated IFN signaling is essential for maintaining the balance of innate immune responses.

The host innate immune responses are often associated with autophagy. For example, TBK1 has a major role in autophagy and mitophagy, primarily in the phosphorylation of autophagy adaptors [16,17]. STING1 (stimulator of interferon response cGAMP interactor 1) can also activate autophagy (a fundamental role in cellular, tissue, and organismal homeostasis and is regulated by the highly conserved *ATG* (autophagy related) genes through a mechanism that is independent of TBK1 [18]. Autophagy, an indispensable biological function that helps to maintain normal tissue homeostasis and metabolic fitness, is classified into macroautophagy, microautophagy, and chaperone-mediated autophagy [19]. Selective macroautophagy/autophagy maintains cellular homeostasis through the lysosomal degradation of specific cellular proteins, viral proteins, mitochondria (mitophagy), or ER (reticulophagy), which plays an important role in host innate immune responses. Autophagy cargo receptors contain the LC3-interacting region/LIR, allowing interaction with Atg8-family members and thus targeting the cargos to phagophores [20]. SQSTM1 is one of the typical autophagy receptors that interact with ubiquitinated substrates via its ubiquitin-associated domain and multimerize via its PB1 domain for transferring to the autophagosome formation site, which is a necessary process for selective autophagic degradation of ubiquitinated substrates [21].

N(6)-methyladenosine (m6A) is one of the most prevalent internal modifications on mRNAs in eukaryotes [22,23]. The major roles of the m6A modification rely on downstream RNA-binding proteins, known as m6A “readers”, that preferentially recognize m6A-modified RNAs. The protein family containing the YTH (YT521-B homology) domain is a group of conserved m6A readers, including the YTH domain family (YTHDF1, YTHDF2 and YTHDF3) and YTH domain-containing proteins (YTHDC1 and YTHDC2). Of them, YTHDF2 is usually known for the degradation of m6A-modified RNAs [24]. Studies have shown that m6A is critical in regulating autophagy by targeting *ATG5* and *ATG7*, the targets of YTHDF2 [25]. In addition, m6A reader YTHDC1 modulates autophagy by targeting SQSTM1 [26]. YTHDF2 can also degrade *STING1* mRNA by recognizing m6A modification to inhibit the innate immune response in teleost fish [27]. However, the multiple functions of YTHDF2 in innate immune response remain unknown.

GTPBP4/NOG1/NGB/CRFG (GTP binding protein 4) is conserved across eukaryotes from yeast to humans and is a novel member of GTPases belonging to the guanine nucleotide-binding proteins family [28]. GTPBP4 locates in the nucleolus and is a multi-functional protein involved in the biogenesis of 60 S ribosomal subunit, DNA mismatch repair system, PKM/PKM2-dependent glucose metabolism, cell cycle, and cancer [29–33]. Although GTPBP4 has multiple biological functions, its role in viral infection is still not fully understood.

In the present study, we investigated the role of GTPBP4 during FMDV infection and identified the function of GTPBP4 to promote FMDV replication in cells and mice. We found that FMDV VP1 interacted with and degraded YTHDF2 in an AKT-MTOR (mechanistic target of rapamycin kinase)-dependent autophagy pathway, resulting in an increase in *GTPBP4* mRNA and protein levels. Increased GTPBP4 inhibited IRF3 binding to the *IFNB/IFN-β* promoter, suppressing FMDV-induced type I interferon production. Our findings show for the first time that FMDV VP1 antagonizes host innate immune responses by promoting autophagy.

## Results

### GTPBP4 promotes FMDV replication in cells and mice

**Figure 1.**
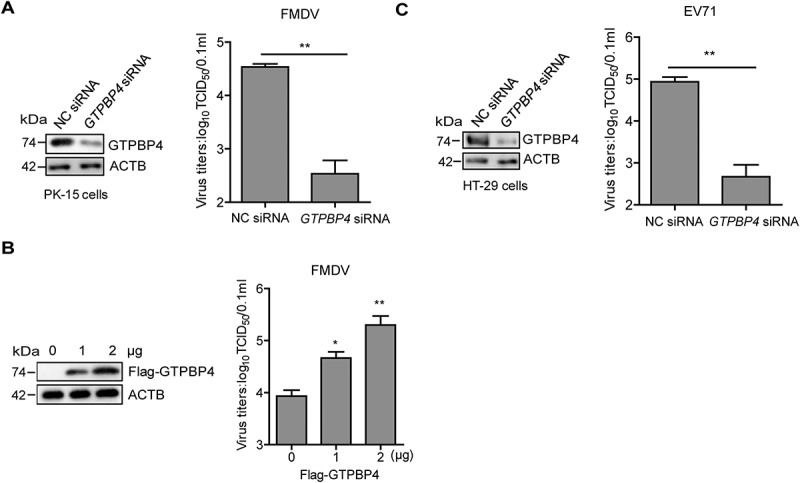
GTPBP4 promotes FMDV replication in cells. PK-15 cells transfected with 150 nM of *GTPBP4* siRNA or NC siRNA were infected with FMDV (MOI 0.1) (A) PK-15 cells transfected with increasing flag-GTPBP4 expression plasmid (0, 1, and 2 μg) were infected with FMDV (MOI 0.1) (B) HT-29 cells transfected with 150 nM of *GTPBP4* siRNA or NC siRNA were infected with EV71 (MOI 1) (C) the viral titers in the supernatant were determined by TCID_50_ assay.

GTPBP4 is a multi-functional protein. Whether GTPBP4 had a regulatory role in FMDV replication required further investigation. PK-15 cells were transfected with asmall interfering RNA (siRNA) targeting *GTPBP4* or negative control (NC). At 36 h post-transfection (hpt), the transfected cells were infected with FMDV. Viral titers were determined by TCID_50_ assay. The results showed that FMDV replication was significantly decreased in *GTPBP4* siRNA-treated cells compared to that in NC siRNA-treated cells (Figure 1A). In addition, overexpression of GTPBP4 significantly promoted FMDV replication in a dose-dependent manner (Figure 1B). Like FMDV, enterovirus 71 (EV71) belongs to the family of *Picornaviridae*. EV71 is an important human pathogen affecting the hand, foot, and mouth disease in infants and young children [34,35]. Therefore, we also evaluated the impact of GTPBP4 on EV71 replication using HT-29 cells that are susceptible to EV71, which indicated that GTPBP4 facilitated the replication of EV71 (Figure 1C).

**Figure 2.**
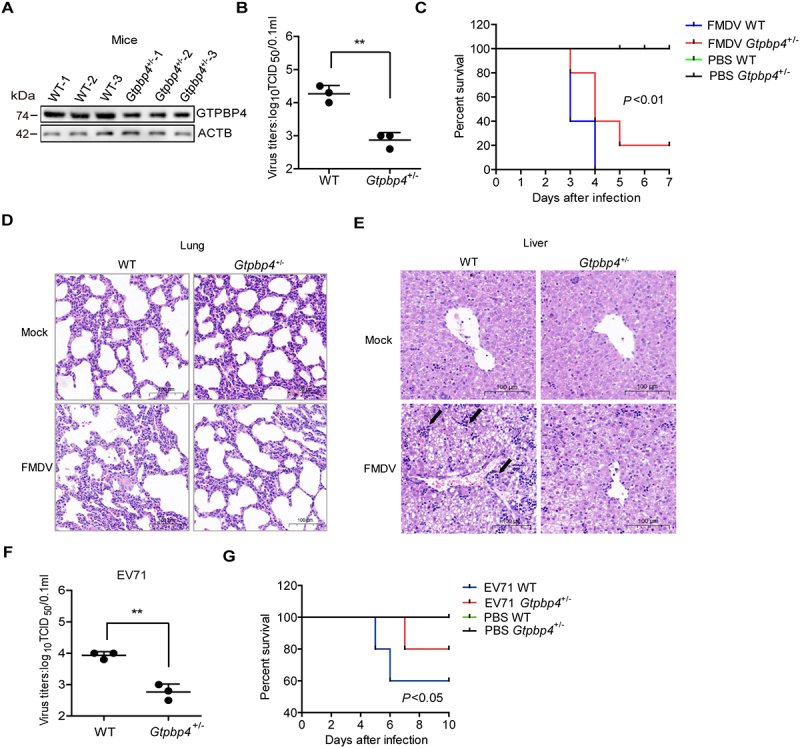
GTPBP4-deficient mice are more resistant to FMDV infection. (A) the expression of GTPBP4 in the carcasses without the head, tail, limbs, and viscera of WT and *Gtpbp4*^±^ mice was detected by western blotting. (B-E) the three-day-old WT and *Gtpbp4*^±^ mice were subcutaneously inoculated with FMDV (10^8^ TCID_50_). FMDV titers in the mice carcasses without the head, tail, limbs, and viscera were determined by TCID_50_ assay (B). The mortality of WT and *Gtpbp4*^±^ mice (*n* = 10) was determined (C). H&E staining was performed for histological examination of the lung (D) and liver (E) of mice. A black arrowhead indicates inflammatory cells in the liver. (F-G) the three-day-old WT and *Gtpbp4*^±^ mice were subcutaneously inoculated with EV71 (10^8^ TCID_50_). The viral titers in the mice carcasses without the head, tail, limbs, and viscera were determined at 2 dpi by TCID_50_ assay. The mortality of mice (*n* = 5) was determined.

To assess the physiologic relevance of GTPBP4 function, we assessed the importance of GTPBP4 in antiviral function in mice. We found that the *gtpbp4*^−/−^ mice are embryonic lethal in the knockout mice experiments. Therefore, the GTPBP4 heterozygous (*Gtpbp4*^±^) mice were used in subsequent experiments. The decrease of GTPBP4 protein in the *Gtpbp4*^±^ mice carcasses without the head, tail, limbs, and viscera was confirmed by western blotting (Figure 2A).

WT and *Gtpbp4*^±^ mice were infected with FMDV, and the titers of FMDV were evaluated and compared, suggesting that the viral titers were significantly decreased in *Gtpbp4*^±^ mice compared to that in WT mice (Figure 2B). The impact of GTPBP4 on FMDV-induced mice mortality was evaluated as well. The WT mice infected with FMDV started to die at 3 d post-infection (dpi) and all mice died by 4 dpi, while the *Gtpbp4*^±^ mice infected by FMDV started to die at 3 dpi and survived 20% at 7 dpi, indicating that GTPBP4 deficiency decelerated FMDV-induced the death of mice (Figure 2C).

To confirm whether GTPBP4 deficiency also reduced tissue injury after FMDV infection, the histological changes in the lung and liver of WT and *Gtpbp4*^±^ mice were detected. There was no histological change in the lung and liver of mock-infected WT and *Gtpbp4*^±^ mice. Pathology examination showed that FMDV infection induced severe alveolar collapse and destroyed lung structure, and less tissue damage morphology was observed in the lung of *Gtpbp4*^±^ mice compared to WT mice (Figure 2D). In addition, FMDV infection induced infiltration of inflammatory cells in the liver, and decreased infiltration of inflammatory cells was observed in the liver of *Gtpbp4*^±^ mice compared to WT mice (Figure 2E). These results indicated that GTPBP4 deficiency protected mice against tissue injury during FMDV infection.

We further assessed the impact of GTPBP4 on EV71 replication in mice. The EV71 titers were significantly decreased in *Gtpbp4*^±^ mice compared to that in WT mice (Figure 2F). EV71-infected WT mice survived 60%, while EV71-infected *Gtpbp4*^±^ mice survived 80% at 10 dpi, suggesting that GTPBP4 deficiency resulted in lower mortality of the mice infected with EV71 (Figure 2G). Taken together, these results indicated that GTPBP4 promotes FMDV and EV71 replication *in vitro* and *in vivo*.

### GTPBP4 involves in FMDV-induced type I interferon production

**Figure 3.**
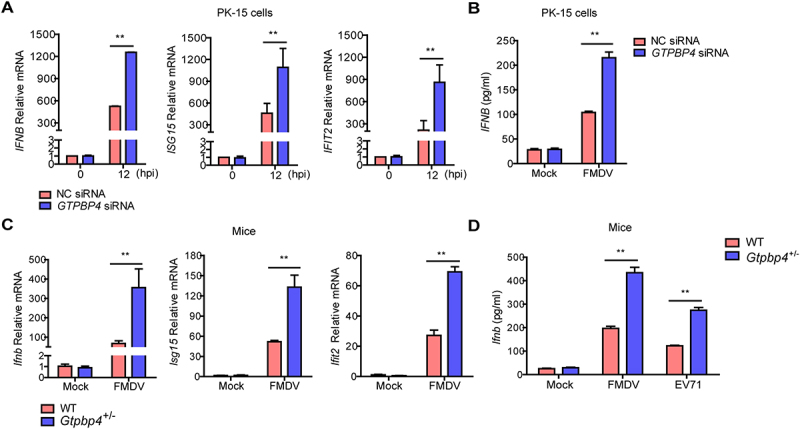
GTPBP4 involves in FMDV-induced type I interferon production. (A) PK-15 cells transfected with 150 nM of *GTPBP4* siRNA or NC siRNA were infected with FMDV (MOI 0.1). The mRNA expression of *IFNB*, *ISG15*, and *IFIT2* was measured by qPCR. The level of *IFNB* protein in the supernatant was detected by ELISA kit (B). (C-D) the three-day-old WT and *Gtpbp4*^±^ mice (*n* = 4) were subcutaneously inoculated with FMDV (10^8^ TCID_50_) or EV71 (10^8^ TCID_50_). The mRNA expression of *Ifnb*, *Isg15*, and *Ifit2* in FMDV-infected mice was measured by qPCR (C). The expression of *ifnb* protein in the mouse serum was detected by ELISA kit (D).

Type I IFN plays an important antiviral role during FMDV infection. Therefore, the impact of GTPBP4 on FMDV-induced type I interferon was detected and compared. PK-15 cells transfected with *GTPBP4* siRNA or NC siRNA were infected with FMDV, and the mRNA expression of *IFNB*, *IFNA1*, *ISG15*, and *IFIT2/ISG54* was measured. Knockdown of *GTPBP4* significantly promoted FMDV-induced *IFNB*, *ISG15*, and *IFIT2* mRNA expression (Figure 3A). However, knockdown of *GTPBP4* did not affect *IFNA1* mRNA expression (Figure S1). In addition, GTPBP4 deficiency significantly enhanced FMDV-induced *IFNB* protein secretion (Figure 3B).

We then detected the impact of GTPBP4 on the expression of *Ifnb*, *Ifna1*, *Isg15*, and *Ifit2* in mice carcasses without the head, tail, limbs, and viscera. The mRNA expression of *Ifnb*, *Isg15*, and *Ifit2*, but not *Ifna1*, was significantly enhanced in the *Gtpbp4*^±^ mice compared to that in the FMDV-infected WT mice (Figure 3C and S1). WT and *Gtpbp4*^±^ mice were infected with FMDV and EV71 to investigate changes in *Ifnb* protein levels. The results showed that the levels of *Ifnb* protein were significantly increased in the serum of *Gtpbp4*^±^ mice compared to that in WT mice (Figure 3D).

Previous studies have shown that TBK1-mediated signal transduction was abnormal in IBRS-2 cells, inhibiting innate immune response-related pathways in IBRS-2 cells during RNA virus infection [36,37]. Therefore, IBRS-2 cells were selected to determine that the promotion of viral replication is indeed due to changes in *IFNB* levels. IBRS-2 cells transfected with increasing Flag-GTPBP4 expression plasmids were infected with FMDV. No significant difference in viral titers was observed (Figure S2), suggesting that GTPBP4 promoted FMDV replication depending on *IFNB*. Taken together, these results indicated that GTPBP4 regulates FMDV and EV71 replication depending on the expression of type I interferon production.

We then tried to explore the mechanisms by which GTPBP4 regulates innate immunity. GTPBP4 was mainly distributed in the nucleus (Figure S3A), which is in accordance with previous studies [38]. Upon RNA virus infection, the interaction between RIGI and viral RNA induces the activation of IRF3 and IRF7, which causes IRF3 and IRF7 to enter the nucleus [39]. Therefore, the impact of GTPBP4 on IRF3- and IRF7-induced innate immune responses was assessed using luciferase reporter assays. GTPBP4 significantly inhibited IRF3- but not IRF7-induced *IFNB* promoter activity (Figure S3B). Thus, we speculated that GTPBP4 antagonized innate immunity by interacting with activated IRF3. As expected, GTPBP4 interacted with p-IRF3 in the context of FMDV infection (Figure S3C). We then identified the region in IRF3 that was essential for GTPBP4-IRF3 interaction. The N-terminal domain (NTD, amino acids 1 to 197) and C-terminal domain (CTD, amino acids 198 to 427) of IRF3 were used to investigate the binding domain of IRF3 [40,41]. The results showed that IRF3 NTD, but not CTD, interacted with GTPBP4 (Figure S3D).

The NTD of IRF3 contains the DNA binding domain [39]. Therefore, we investigated the impact of GTPBP4 on the DNA binding ability of IRF3. The effect of GTPBP4 on IRF3 binding onto promoter was analyzed using chromatin immunoprecipitation (ChIP) assay and qPCR. The levels of immunoprecipitated DNA were normalized to the input DNA levels. The results showed that overexpression of GTPBP4 inhibited the interaction between IRF3 and the *IFNB* promoter (Figure S3E), and GTPBP4 deficiency promoted the binding of IRF3 and the *IFNB* promoter (Figure S3F). Meanwhile, our data indicated that GTPBP4 also blocked EV71-induced interaction between IRF3 and *IFNB* promoter in HT-29 cells (Figure S3G). These results indicated that GTPBP4 inhibits *IFNB* expression by impairing the DNA binding ability of IRF3.

### FMDV infection promotes the expression of GTPBP4

**Figure 4.**
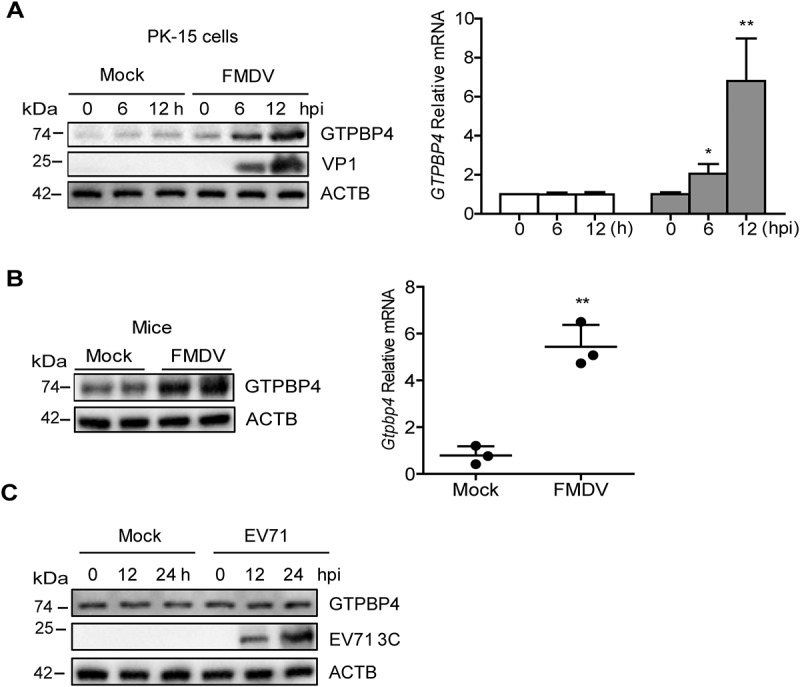
FMDV infection promotes the expression of GTPBP4. (A) PK-15 cells were mock-infected and infected with FMDV (MOI 0.1) for 0, 6, and 12 h. The expression of *GTPBP4* protein and mRNA was detected by western blotting and qPCR, respectively. (B) the three-day-old WT mice were subcutaneously inoculated with or without FMDV (10^8^ TCID_50_) for 2 d. The expression of *Gtpbp4* protein and mRNA in the mice carcasses without the head, tail, limbs, and viscera was detected by western blotting and qPCR, respectively. (C) HT-29 cells were mock-infected or infected with EV71 (MOI 1) for 0, 12, and 24 h. The abundance of GTPBP4 protein was determined by western blotting.

The regulatory relationship between viruses and GTPBP4 is unclear. To further explore the potential role of GTPBP4 in picornavirus infection, we investigated the state of GTPBP4 in picornavirus-infected cells. PK-15 cells were infected with FMDV and the dynamics of GTPBP4 were determined. The results showed that GTPBP4 transcription was significantly upregulated as the infection progressed (Figure 4A). We also detected the abundance of GTPBP4 protein in FMDV-infected cells. The protein level of GTPBP4 gradually increased as infection progressed (Figure 4A). Meanwhile, as a control, there were no significant changes in *GTPBP4* mRNA and protein levels in mock-infected cells.

To further confirm the impact of FMDV infection on GTPBP4 expression, mice were mock-infected and infected with FMDV and the expression of *Gtpbp4* mRNA and protein was detected. Again, FMDV infection promoted the levels of *Gtpbp4* mRNA and protein in mice (Figure 4B). However, our data showed that EV71 infection did not affect the expression of GTPBP4 in cells (Figure 4C). Taken together, these results indicated that FMDV infection promoted the expression of GTPBP4 protein.

### FMDV VP1 plays a role in promoting the expression of GTPBP4

**Figure 5.**
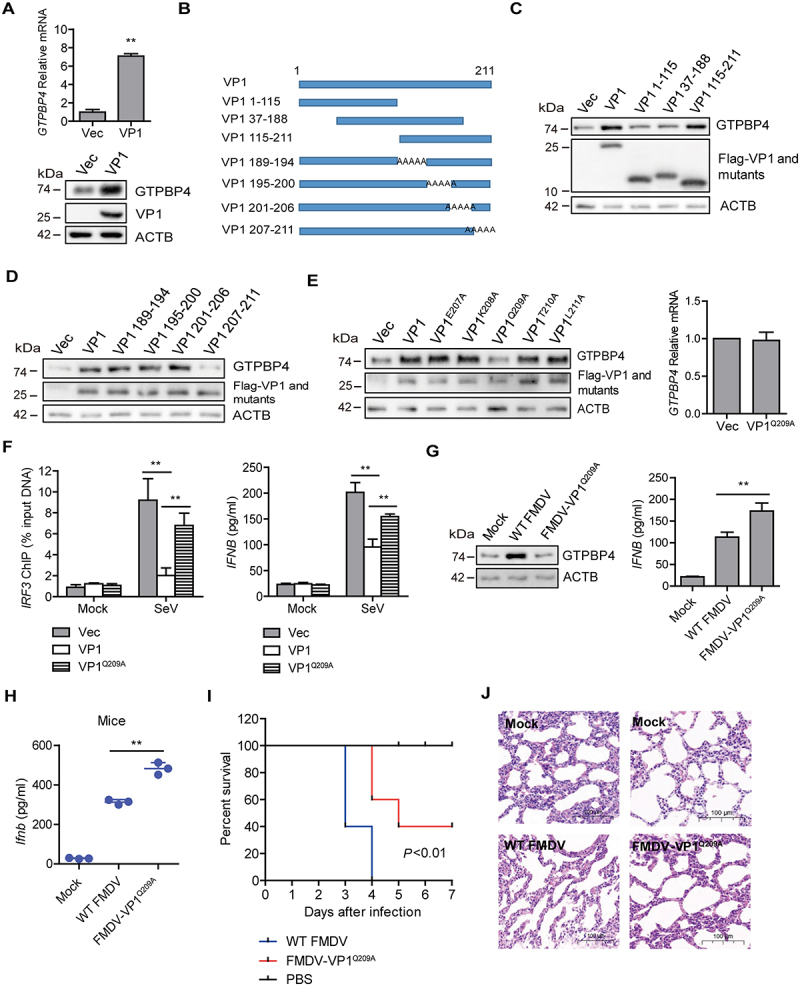
FMDV VP1 was responsible for the increase of GTPBP4. (A) PK-15 cells were transfected with 2 μg of plasmids expressing flag-VP1 proteins. The expression of *GTPBP4* protein and mRNA was detected by western blotting and qPCR, respectively. (B) schematic representation showing a series of flag-tagged truncated VP1 mutants. (C-E) PK-15 cells were transfected with 2 μg of empty vector, flag-VP1- or the indicated flag-VP1-mutants-expressing plasmids. At 24 hpt, the expression of GTPBP4 protein was determined by western blotting. The effect of Flag-VP1^Q209A^ on *GTPBP4* mRNA expression was detected by qPCR. (F) PK-15 cells transfected with empty vector, flag-VP1, or Flag-VP1^Q209A^ expression plasmid were infected with SeV for 12 h. Chromatin was immunoprecipitated with an anti-IRF3 antibody. The impact of VP1 on IRF3 binding onto *IFNB* promoter was analyzed by quantitative ChIP assay (left). The abundance of the immunoprecipitated DNA was normalized to the input DNA levels. The expression of *IFNB* protein in the supernatant was detected by ELISA kit (right). (G) PK-15 cells were mock-infected and infected with WT FMDV or FMDV-VP1^Q209A^ for 8 h. The expression of GTPBP4 protein was detected by western blotting. The level of *IFNB* protein in the supernatant was detected by ELISA kit. (H-J) the three-day-old WT mice were subcutaneously inoculated with WT FMDV (10^8^ TCID_50_) or FMDV-VP1^Q209A^ (10^8^ TCID_50_). The expression of *Ifnb* protein in the mice serum was detected by ELISA kit (H). The mortality of WT FMDV- and FMDV-VP1^Q209A^-infected mice (*n* = 10) was determined (I). H&E staining was performed for histological examination of the lung in mice (J).

To investigate the viral proteins that may be responsible for the increase of GTPBP4, PK-15 cells were transfected with plasmids expressing different Flag-tagged viral proteins. The expression of GTPBP4 was determined by western blotting, suggesting that overexpression of VP1, but not other proteins, enhanced GTPBP4 protein abundance (Figure 5A and S4). Overexpression of VP1 also promoted the mRNA expression of *GTPBP4* (Figure 5A). To investigate a possible interaction between GTPBP4 and VP1, PK-15 cells were mock-infected or infected with FMDV. The cell lysates were immunoprecipitated with anti-GTPBP4 or anti-VP1 antibodies and subjected to immunoblotting analysis. GTPBP4 did not pull down VP1, and VP1 also did not pull down GTPBP4, indicating no interaction between GTPBP4 and VP1 in the context of viral infection (Figure S5).

To confirm the functional sites of VP1 that were essential for the increase of GTPBP4, a series of truncated or site-mutation mutant constructs of FMDV VP1 were generated and used for detailed analyses (Figure 5B). Flag-VP1 or truncated Flag-VP1 mutants were transfected into PK-15 cells, and the abundance of GTPBP4 was determined by western blotting. The results showed that the 189–211 amino acid region of VP1 was essential for enhancing GTPBP4 protein (Figure 5C). The functional sites in the carboxyl-terminal 189–211 amino acid region were subsequently analyzed. A series of plasmids expressing carboxyl terminal mutants of Flag-VP1 were transfected into PK-15 cells, and the expression of GTPBP4 was detected by western blotting. The results showed that the 209th aa site in VP1 was critical for promoting GTPBP4 expression (Figure 5D,E). The 209th site of VP1 was also necessary to promote the mRNA expression of *GTPBP4* (Figure 5E). These results indicated that the 209th site of VP1 was responsible for the increase of GTPBP4. We then mapped the structure and functional regions of VP1 using the iterative threading assembly refinement (I-TASSER) server and consurf analysis tools. The 209th site of VP1 is mainly distributed on the surface of the virus (Figure S6), revealing the importance of this site.

As described above, GTPBP4 impaired the DNA binding ability of IRF3, and FMDV VP1 promoted GTPBP4 protein expression. Therefore, the impact of VP1 on the DNA binding ability of IRF3 was detected using ChIP assay and qPCR. Overexpression of VP1 inhibited the interaction between IRF3 and the *IFNB* promoter, while overexpression of VP1^Q209A^ significantly restored this inhibitory effect (Figure 5F, left panel). Furthermore, the impact of VP1 on SeV-induced *IFNB* expression was determined and compared. Overexpression of VP1 significantly decreased SeV-induced *IFNB* protein secretion, and the expression of *IFNB* was significantly enhanced in Flag-VP1^Q209A^-transfected cells compared to that in Flag-VP1-transfected cells (Figure 5F, right panel). These results suggested that FMDV VP1 could inhibit the DNA binding ability of IRF3, and the 209th site of VP1 was responsible for this effect.

To further confirm this site’s role in promoting GTPBP4 expression, a recombinant FMDV was tried to be rescued by introducing single-site mutation Q209A. The recombinant wildtype FMDV was used as the parental virus (WT FMDV). The Q209A mutant FMDV was successfully rescued (named FMDV-VP1^Q209A^). The expression of GTPBP4 and *IFNB* protein in WT FMDV- and FMDV-VP1^Q209A^-infected cells was evaluated and compared. WT FMDV induced the increase of GTPBP4, while FMDV-VP1^Q209A^ lost its ability to promote GTPBP4 expression (Figure 5G). Meanwhile, the expression *IFNB* was significantly enhanced in FMDV-VP1^Q209A^-infected cells compared to that in WT FMDV -infected cells (Figure 5G), confirming the effect of the 209th site of VP1 on GTPBP4 and *IFNB* expression.

Subsequently, we further detected the expression of *Ifnb* and viral yield in WT FMDV- and FMDV-VP1^Q209A^-infected suckling mice. Mice were mock-infected or infected with WT FMDV and FMDV-VP1^Q209A^, the levels of *Ifnb* protein in serum were detected by ELISA. Again, the levels of *Ifnb* protein were significantly increased in FMDV-VP1^Q209A^-infected mice compared to that in WT FMDV -infected mice (Figure 5H). The mortality of WT FMDV- and FMDV-VP1^Q209A^-infected mice was also evaluated. WT FMDV-infected mice started to die at 3 dpi and all mice died by 4 dpi, while FMDV-VP1^Q209A^-infected mice started to die at 4 dpi and survived 40% at 7 dpi (Figure 5I), indicating that FMDV-VP1^Q209A^ infection decelerated the death of mice. Furthermore, pathology examination showed less tissue damage morphology in the lung of FMDV-VP1^Q209A^-infected mice compared to WT FMDV -infected mice (Figure 5J). This illustrated that the Q209 point mutation within VP1 of FMDV attenuated pathogenicity. Taken together, these results indicated that FMDV VP1 was essential for the increase of GTPBP4, and the 209th site of VP1 was responsible for this effect.

### FMDV VP1 promotes GTPBP4 expression by degrading YTHDF2

The m6A readers, YTH domain proteins including YTHDF1, YTHDF2, YTHDF3, and YTHDC2 that mainly located in the cytoplasm, which play important roles in accelerating metabolism of m6A-modified mRNAs [42]. To investigate the mechanism of VP1 promoting GTPBP4 expression, specific siRNAs were used to knock down *YTHDF1*, *YTHDF2*, *YTHDF3*, and *YTHDC2*, and the effect of these proteins on *GTPBP4* mRNA expression was detected. The results showed that knockdown of *YTHDF2* promoted *GTPBP4* mRNA expression, while knockdown of *YTHDF1*, *YTHDF3*, and *YTHDC2* did not affect the expression of GTPBP4 (Figure S7).

**Figure 6.**
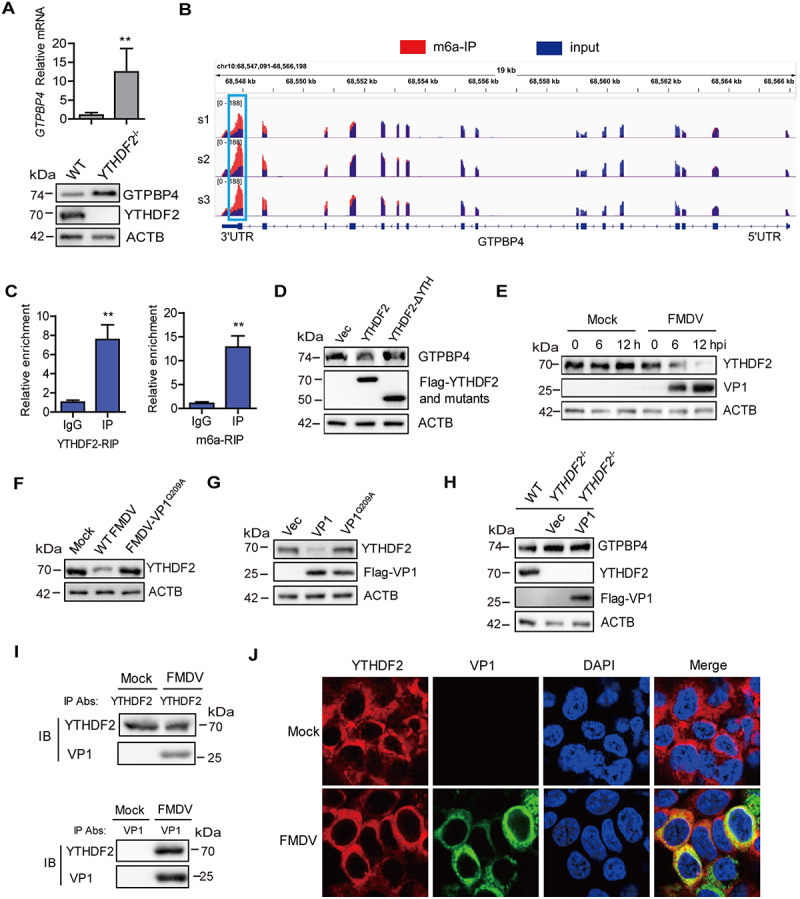
FMDV VP1 enhances GTPBP4 expression by degrading YTHDF2. (A) the protein and mRNA levels of *GTPBP4* in the WT and *YTHDF2*^−/−^ PK-15 cells were detected by western blotting and qPCR, respectively. (B) total RNA was isolated from PK-15 cells. The RNA was immunoprecipitated with m6A-specific antibody. The immunoprecipitated RNA was reverse-transcribed to cDNA. The methylation of GTPBP4 was determined by paired-end sequencing. s: sample. (C) PK-15 cells transfected with 5 μg of flag-YTHDF2 expression plasmid were collected and immunoprecipitated using anti-IgG or anti-flag antibody (left). Total RNA was immunoprecipitated with m6A-specific antibody (right). The immunoprecipitated RNA was reverse-transcribed to cDNA. The levels of methylated *GTPBP4* mRNA were detected by qPCR. (D) PK-15 cells were transfected with 2 μg of flag-YTHDF2 or flag-YTHDF2-ΔYTH expression plasmid for 24 h. The protein expression of GTPBP4 and YTHDF2 was detected by western blotting. (E-F) PK-15 cells were mock-infected or infected with WT FMDV or FMDV-VP1^Q209A^. The expression of YTHDF2 protein was detected by western blotting. (G) PK-15 cells were transfected with 2 μg of flag-VP1 or Flag-VP1^Q209A^ expression plasmid for 24 h. The protein expression of YTHDF2 was detected by western blotting. (H) *YTHDF2*^−/−^ cells were transfected with 2 μg of empty vector or flag-VP1 expression plasmid for 24 h. The expression of GTPBP4 and YTHDF2 was detected by western blotting. (I-J) PK-15 cells were mock-infected or infected with FMDV (MOI 0.1) for 6 h. The cell lysates were immunoprecipitated with anti-YTHDF2 or anti-VP1 antibodies. The antibody-antigen complexes were analyzed by the indicated antibodies (I). The intracellular localization of YTHDF2 and VP1 was detected by IFA using anti-YTHDF2 and anti-VP1 antibodies (J).

YTHDF2 was the first discovered and most efficient m6A “reader” and it could regulate mRNA degradation [43]. Therefore, we further investigated the impact of YTHDF2 on GTPBP4 expression using *YTHDF2* knockout (*YTHDF2*^−/−^) PK-15 cells. The protein and mRNA levels of *GTPBP4* were enhanced in the *YTHDF2*^−/−^ cells compared to that in WT cells (Figure 6A). The knockout of *YTHDF2* was confirmed by western blotting (Figure 6A). YTHDF2 degrades mRNA by interacting with m6A in mRNA [44]. Thus, we speculated that *GTPBP4* mRNA can be methylated. To test this hypothesis, we obtained a more confident set of m6A-bound transcripts by performing m6A RNA immunoprecipitation (RIP) followed by sequencing. Based on the m6A RIP sequencing (MeRIP-seq) data (Table S1), we found that GTPBP4 transcripts contained m6A modification that mainly at the 3′UTR region (Figure 6B), indicating that GTPBP4 could be regulated at the epitranscriptomic level. RIP using either YTHDF2 or m6A antibody followed by quantitative PCR (qPCR) further confirmed that GTPBP4 transcript is indeed methylated and bound by YTHDF2 (Figure 6C). YTHDF2 has a YTH domain that is essential for methylated mRNA degradation [27]. For a further functional experiment, we truncated the YTH domain to construct the mutant (YTHDF2-ΔYTH), as previously described [43]. As shown in Figure 6D, overexpression of YTHDF2 can reduce the expression of GTPBP4 protein, whereas overexpression of YTHDF2-ΔYTH did not affect GTPBP4 expression, suggesting that loss of the YTH domain of YTHDF2 completely abolished its function in inhibiting GTPBP4, which further confirmed that YTHDF2 does regulate the expression of GTPBP4 through methylation.

We then investigated the regulatory relationship between FMDV and YTHDF2. PK-15 cells were infected with WT FMDV or FMDV-VP1^Q209A^, and the expression of YTHDF2 was detected by western blotting. The results showed that the protein level of YTHDF2 gradually decreased as infection progressed (Figure 6E), and FMDV-VP1^Q209A^ lost its ability to reduce YTHDF2 expression (Figure 6F). The effect of VP1 on the expression of YTHDF2 was further confirmed. Overexpression of VP1, but not VP1^Q209A^, induced the reduction of YTHDF2 (Figure 6G), which is consistent with the site where VP1 promoted GTPBP4 expression, suggesting that VP1 May promote GTPBP4 expression through YTHDF2. To prove this conclusion, we transfected the empty vector and VP1 plasmids into *YTHDF2*^−/−^ cells, and the expression of GTPBP4 protein was confirmed by western blotting. The level of GTPBP4 protein was increased in the *YTHDF2*^−/−^ cells compared to that in WT cells, whereas overexpression of VP1 no longer promoted GTPBP4 protein abundance (Figure 6H), demonstrating that VP1 promoted GTPBP4 expression depending on YTHDF2.

To investigate the interaction between YTHDF2 and VP1, PK-15 cells infected with FMDV were immunoprecipitated with anti-YTHDF2 or anti-VP1 antibodies and subjected to immunoblotting analysis. YTHDF2 pulled down VP1 and VP1 pulled down YTHDF2 as well, suggesting an interaction between YTHDF2 and VP1 in the context of viral infection (Figure 6I). The interaction between YTHDF2 and VP1 was further detected by IFA (indirect immunofluorescence assay). Although VP1 degraded YTHDF2, the interaction was observed by IFA (Figure 6J), which further demonstrated that YTHDF2 interacted with VP1. Taken together, these results indicated that FMDV VP1 promoted GTPBP4 expression by inducing the degradation of YTHDF2.

## FMDV VP1 promotes YTHDF2 degradation through an AKT-MTOR-dependent autophagy pathway

**Figure 7.**
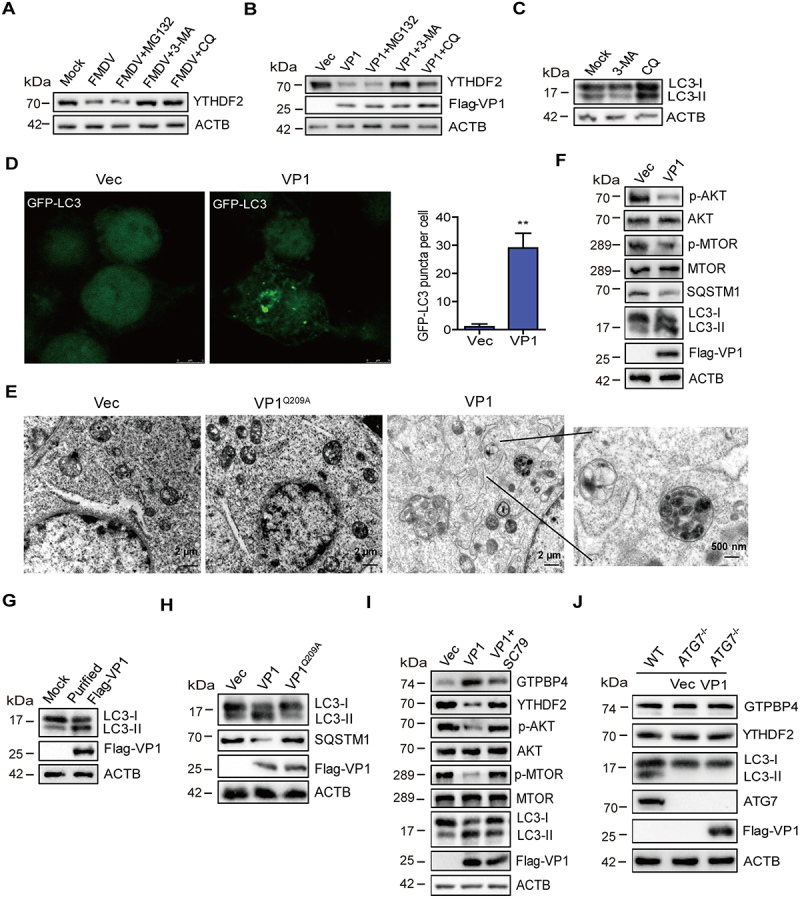
FMDV VP1 promotes YTHDF2 degradation through an AKT-MTOR-dependent autophagy pathway. (A) PK-15 cells were infected with FMDV (MOI 0.1). At 1 hpi, the cells were maintained in the fresh medium in the presence or absence of MG132 (20 μM), 3-MA (1 mM), or CQ (100 μM). At 12 hpi, the abundance of YTHDF2 was determined by western blotting. (B) PK-15 cells were transfected with 2 μg of flag-VP1-expressing plasmid. At 6 hpt, the cells were maintained in the fresh medium in the presence or absence of MG132 (20 μM), 3-MA (1 mM), or CQ (100 μM) for 18 h. Expression of YTHDF2 protein was determined by western blotting. (C) PK-15 cells were incubated with 3-MA (1 mM) or CQ (100 μM) for 18 h. Expression of LC3-I and LC3-II protein was determined by western blotting. (D) PK-15 cells were transfected with 2 μg of GFP-LC3 and flag-VP1-expressing plasmids for 24 h. The autophagosomes were detected using a confocal laser scanning microscope. (E) PK-15 cells were transfected with 2 μg of empty vector or flag-VP1-expressing plasmids for 24 h. The samples were analyzed by transmission electron microscopy to show autophagosomes. (F-H) PK-15 cells were transfected with 2 μg of empty vector, flag-VP1-, or Flag-VP1^Q209A^-expressing plasmids for 24 h (F and H). PK-15 cells were incubated with the purified flag-VP1 (150 μg/mL) (G). The cells were collected and subjected to western blotting analysis. (I) PK-15 cells were transfected with 2 μg of empty vector or flag-VP1-expressing plasmids for 24 h and maintained in the presence or absence of SC79 (10 μM) for 12 h. The cells were collected and subjected to western blotting analysis. (J) *ATG7*^−/−^ cells were transfected with 2 μg of empty vector or flag-VP1-expressing plasmids for 24 h. The expression of GTPBP4, YTHDF2, and LC3 was detected by western blotting.

Proteasomes and autophagy-lysosome pathways are two major intracellular protein degradation pathways in cells [45,46]. To assess whether these pathways were associated with FMDV-induced reduction of YTHDF2, the autophagy inhibitors CQ (chloroquine diphosphate) and 3-MA and proteasome inhibitor MG132 were used to block these pathways. PK-15 cells infected with FMDV were maintained in the presence or absence of these inhibitors. The expression of YTHDF2 was determined by western blotting. FMDV-induced decrease of YTHDF2 was inhibited by CQ and 3-MA but not MG132 (Figure 7A). The effect of the inhibitors on VP1-induced reduction of YTHDF2 was evaluated as well. Incubation of VP1 overexpressing cells with CQ or 3-MA reversed VP1-induced YTHDF2 degradation, while treatment with MG132 did not affect the degradation of YTHDF2 (Figure 7B). The efficacy of 3-MA and CQ was verified (Figure 7C). This indicated that both FMDV- and VP1-induced reduction of YTHDF2 were dependent on the autophagy pathway.

MAP1LC3/LC3 (microtubule associated protein 1 light chain 3) is expressed in most cell types as a full-length cytosolic protein. Upon induction of autophagy, LC3 is proteolytically cleaved by ATG4 to generate LC3-I, which can further generate processed LC3-II, causing LC3-II to bind to the autophagosome, where it plays a role in selecting cargo for degradation [47]. To evaluate the effect of VP1 on the induction of autophagy, PK-15 cells expressing GFP-LC3 along with empty vector or VP1 were detected using a confocal laser scanning microscope to observe autophagosomes. The results showed that the expression of VP1 significantly increased the accumulation of fluorescent puncta of LC3 (Figure 7D), indicating the induction of autophagy. Electron microscopy is one of the most commonly used and effective methods for detecting autophagy. Thus, the impact of VP1 on autophagy was examined by electron microscopy. As shown in Figure 7E, compared with the control, the expression of VP1 induced more vesicles, which had typical characteristics of autophagic vesicles. We then analyzed the mechanism by which VP1 induces autophagy. The MTOR signaling is a central regulator of autophagy, and AKT plays an important role in the maintenance of the activity of MTOR [48,49]. Therefore, the impact of VP1 on the expression of AKT, MTOR, and LC3 was determined and compared by western blotting. The expression of VP1 decreased the phosphorylation of AKT and MTOR, resulting in the increased expression of LC3-II (Figure 7F). Apart from LC3-II, the level of SQSTM1 also can be used to evaluate autophagy. SQSTM1 is selectively incorporated into autophagosomes by binding to LC3 and can be efficiently degraded through autophagy [14,50]. Thus, the intracellular SQSTM1 level was negatively correlated with autophagy activity. Our data also indicated the reduction of SQSTM1 level in the VP1-transfected cells compared to that in empty vector-transfected cells (Figure 7F). In addition, FMDV infection did not induce the cleavage of SQSTM1 (Figure S8A), which is inconsistent with previous results that Seneca valley virus targeted the receptor SQSTM1 for cleavage [21]. To further evaluate the impact of VP1 on autophagy, eukaryotic expression of Flag-VP1 was purified. The results showed that the purified Flag-VP1 also promoted the expression of LC3-II (Figure 7G). Further study showed that VP1 inhibited the phosphorylation of ULK1 S757 (Figure S8B), suggesting that the low MTOR activity could not phosphorylate ULK1 S757, resulting in autophagy initiation.

To detect the effect of empty capsids on autophagy, we prepared empty capsids containing FMDV structural proteins (VP0, VP3, and VP1), as described previously [51]. Autophagy was induced by the addition of empty capsids (Figure S8C). The impact of FMDV on autophagy was further evaluated using UV inactivation of FMDV, suggesting that UV-WT FMDV or UV-FMDV-VP1^Q209A^ induced the expression of LC3-II (Figure S8D). These results indicated that FMDV replication was not required to induce autophagy. Subsequently, we identified the site where VP1 induces autophagy, which indicated that overexpression of VP1^Q209A^ lost the ability to decrease the phosphorylation of AKT and MTOR (Figure S8E) and induce the increased of LC3-II and autophagy (Figure 7E,H), which is consistent with the site where VP1 degraded YTHDF2 and promoted GTPBP4 expression. These results indicated the pathway and functional site that VP1 induces autophagy.

SC79, a specific AKT activator, was used to inhibit AKT membrane translocation and promote the phosphorylation of AKT [52]. To further determine that VP1 degraded YTHDF2 through the AKT-MTOR-dependent autophagy pathway, we detected the effect of VP1 on YTHDF2 expression in SC79-treated cells. As expected, the treatment of SC79 promoted the phosphorylation of AKT and MTOR. Compared with control cells, VP1 could degrade the expression of YTHDF2, but in SC79-treated cells, the VP1-induced decrease in YTHDF2 was recovered by AKT activation. Meanwhile, VP1 reduced the ability to promote GTPBP4 expression in SC79-treated cells (Figure 7I). The results confirmed that VP1 regulated the expression of YTHDF2 and GTPBP4 through the AKT-MTOR-dependent autophagy pathway.

*ATG5* and *ATG7* are well-known genes regulating autophagy [47]. Therefore, PK-15-*ATG5* knockout (*ATG5*^−/−^) and PK-15-*ATG7* knockout (*ATG7*^−/−^) cell lines were used further to investigate the impact of autophagy on YTHDF2 degradation. *ATG7*^−/−^ cells were transfected with empty vector or Flag-VP1 expression plasmid, and the expression of YTHDF2 and GTPBP4 was detected. The expression of VP1 in the *ATG7*^−/−^ cells could no longer induce the degradation of YTHDF2 and the increase of GTPBP4 (Figure 7J). Similar results were also observed in *ATG5*^−/−^ cells (Figure S8F). *ATG5* and *ATG7* knockout resulted in VP1 losing its ability to promote GTPBP4 expression. Logically, the replication of FMDV in *ATG5* and *ATG7* knockout cells is decreased. As expected, the knockout of *ATG5* or *ATG7* reduced FMDV replication in PK-15 cells (Figure S8G). Taken together, these results indicated that VP1 induced YTHDF2 autophagic degradation in an AKT-MTOR-dependent autophagy pathway.

## Discussion

Autophagy is often associated with viral replication. Although studies have shown that FMDV utilizes autophagy to facilitate its replication, the detailed mechanism still needs to be understood. The present study found that GTPBP4, a novel member of GTPases belonging to the guanine nucleotide-binding proteins family, inhibited IRF3 binding to the *IFNB* promoter. FMDV VP1 degraded YTHDF2 in an AKT-MTOR-dependent autophagy pathway to increase *GTPBP4* mRNA and protein levels, suppressing FMDV-induced type I interferon production. Our data establish a new connection between autophagy and innate immune responses during FMDV infection.

TLR (toll like receptor)-, rig like receptor (RLR)-, nod like receptor (NLR)-, or DNA receptor CGAS-mediated innate immune response forms the first line of defense that protects hosts from invasion by viruses. After viral infection, these receptors recruit downstream molecules, including MAVS, TBK1, or RIP2 to activate IRF3, a common molecule in all innate immune signaling pathways. The study of the mechanisms of innate immune response could contribute to better disease control. In the present study, we investigated for the first time the functions of GTPBP4 during FMDV infection and provided evidence that heterozygous knockout of the *Gtpbp4* gene in mice promotes FMDV-induced *Ifnb* protein and renders the mice more resistant to FMDV infection. Furthermore, FMDV VP1 protein promotes the expression of GTPBP4 to inhibit the innate immune responses by the autophagy degradation of YTHDF2. This finding establishes a key role for GTPBP4 during FMDV infection. GTPBP4 involves many biological processes, including 60 S ribosomal subunit biogenesis, cell cycle, and DNA mismatch repair system [53–55]. The knockout of *GTPBP4* promotes cell cycle arrest in the G_2_/M period [28,30], which may be why *gtpbp4* knockout mice did not survive.

IRF3 is a key transcription factor that plays a key role in the induction of *IFNB* and is essential for the expression of many antiviral genes [56]. Upon viral stimulation, cytoplasmic IRF3 is phosphorylated, forms dimers, and enters the nucleus, where IRF3 interacts with the promoters of *IFNB*, *IFIT2*, or *IFIT1*, leading to the transcription of type I interferon and the downstream ISGs [57]. A variety of regulation mechanisms targeting IRF3 have been identified. For instance, cell growth-regulating nucleolar protein LYAR antagonizes innate immune responses by inhibiting the DNA binding ability of IRF3 [13]; JMJD6 (jumonji domain containing 6, argining demethylase and lysine hydroxylase) negatively regulates RNA viruses-induced antiviral signaling by promoting K48 ubiquitination of IRF3 [58]; PRMT6 negatively regulates innate immunity by inhibiting phosphorylation of IRF3 [59]. In addition, some viruses also regulate the function of IRF3, such as, the nonessential accessory protein ML of Thogoto virus antagonizes the host innate immune responses by blocking the interaction between IRF3 and CREB-binding protein [60]; Ebola virus suppresses the host’s innate immune response by blocking dimerization and phosphorylation of IRF3 [61]; Seneca valley virus abrogates the IRF3-mediated innate immune response by degrading IRF3 [62]; FMDV VP1 target the MAVS to inhibit type I interferon signaling and VP1 E83K is essential for this effect [7]; FMDV VP1 antagonizes TPL2-mediated activation of the IRF3 signaling pathway to facilitate the virus replication [8]; and FMDV VP1 inhibits *IFNB* signaling pathway by blocking the phosphorylation and nuclear translocation of IRF3 [63]. Here, our results showed that FMDV VP1 impairs the DNA binding ability of IRF3 by promoting the expression of GTPBP4, resulting in the inhibition of *IFNB* production, and VP1 Q209 is essential for this inhibitory effect, revealing the importance of this site. This finding uncovers a novel function of GTPBP4 and broadens the regulation mechanisms targeting IRF3 during FMDV infection. Activation of IRF3 involves multiple processes including IRF3 protein expression, IRF3 phosphorylation and nuclear translocation, and the binding of IRF3 with promoters [10]. As a whole, FMDV VP1 negatively regulates IRF3 function at multiple processes.

FMDV promotes the expression of GTPBP4 by degrading YTHDF2. The expression of GTPBP4 and YTHDF2 proteins directly affects their function. To date, the regulation of GTPBP4 protein expression by viruses has not been reported. Our results show for the first time that viruses can regulate GTPBP4 expression. FMDV, but not EV71, induced the expression of GTPBP4, which may be due to differences in viral sequence or structure and deserves further study. The regulation of YTHDF2 protein expression through various mechanisms is sporadically reported. Histone lactylation promotes oncogenesis by facilitating the expression of YTHDF2 in ocular melanoma [64]. The protein levels of YTHDF2 did not change significantly in A549 cells during influenza virus infection [65]. Here, our data indicated that FMDV infection inhibited the expression of YTHDF2 by autophagy pathway, revealing a novel regulation mechanism of YTHDF2 expression. YTHDF2 can promote the degradation of large amounts of methylated mRNA, including *STING1* [27], *CDKN1B* [66], *UBXN1* [67], and *EGFR* [68]. Our results confirmed for the first time that *GTPBP4* mRNA can undergo methylation and showed that YTHDF2 degraded the mRNA of *GTPBP4*, revealing a new function of YTHDF2 protein. FMDV infection induces the increase of mRNA of many proteins, including *NOD2*, *RIP2*, *RIGI*, and *LGP2*, but the involved mechanism has not been clarified [69–71]. Here, we identified a lot of proteins that can be methylated in PK-15 cells (Table S1), showing that NOD2, RIP2, RIGI, and LGP2 proteins can also be methylated. The decrease of YTHDF2 induced by FMDV may be responsible for the increase in the mRNA of these proteins.

FMDV induces the reduction of YTHDF2 expression by the autophagy pathway. Autophagy begins with the formation of a phagophore. The phagophore expands to engulf intracellular cargo, including protein aggregates, organelles, and ribosomes, resulting in cargo sequestration using a double-membraned autophagosome. The loaded autophagosome matures through fusion with the lysosome, facilitating lysosomal acid proteases’ degradation of autophagosomal contents [47]. MTOR-mediated signaling is the main gateway to initiate autophagy [49]. Autophagy is induced by hypoxia and low cytosolic ATP levels that feed through AMP-kinase to inhibit MTOR activity, resulting in the formation of the ULK1-containing pro-autophagic complex, which ultimately ensures the formation of autophagosomes [47]. The ubiquitin-like systems are key to autophagy at the ATG12–ATG5 conjugation step and at the LC3 processing step [72]. Selective autophagy, an important autophagy pathway, is mediated by cargo receptors including SQSTM1, NBR1, or TAX1BP1, which link substrate proteins to LC3 on the autophagosome, where they are packaged into the autophagosome and degraded by lysosome [73]. Many viral proteins can degrade host proteins through autophagy, thereby regulating viral replication [21,45,74–76]. FMDV capsid protein VP2 activates the cellular EIF2S1-ATF4 pathway and induces autophagy by HSPB1 protein, and viral replication is not required for FMDV-induced autophagy [77], and FMDV structural protein VP3 degrades HDAC8 in an AKT-MTOR-ATG5-dependent autophagy pathway to facilitate viral replication [78]. The VP2- and VP3-induced autophagy explains why UV-FMDV-VP1^Q209A^ can induce autophagy. Our results showed that FMDV structural protein VP1 degrades YTHDF2 in an AKT-MTOR-dependent autophagy pathway to promote GTPBP4 expression for viral replication, which follows previously reported results that VP1 antagonizes the AKT signaling pathway [6].

The MTOR-mediated signaling is associated with multiple downstream pathways including mRNA translation, metabolism, and protein turnover (autophagy, apoptosis, or ubiquitin-proteasome system) [49]. Our previous results have shown that FMDV regulates viral RNA translation by the MTOR-mediated pathway [79]. FMDV VP1 induces apoptosis via the AKT signaling pathway [6]. Our data also revealed that VP1 induced autophagy and regulated expression of SIRT3 (sirtuin 3) and HIF1A (hypoxia inducible factor 1 subunit alpha) proteins (Figure S8B) that are downstream molecules of MTOR and involved in energy metabolism [74]. These results suggested that FMDV or VP1 may regulate multiple downstream pathways of MTOR, affecting intracellular autophagy, protein synthesis, or energy metabolic processes. These downstream pathways may work together to regulate the host’s innate immune responses. In the present study, we focused on elucidating the mechanism by which VP1 regulates GTPBP4-mediated innate immune responses through the AKT-MTOR-ULK1-dependent autophagy pathway. The impact of other downstream pathways of MTOR on innate immunity deserves further study.

**Figure 8.**
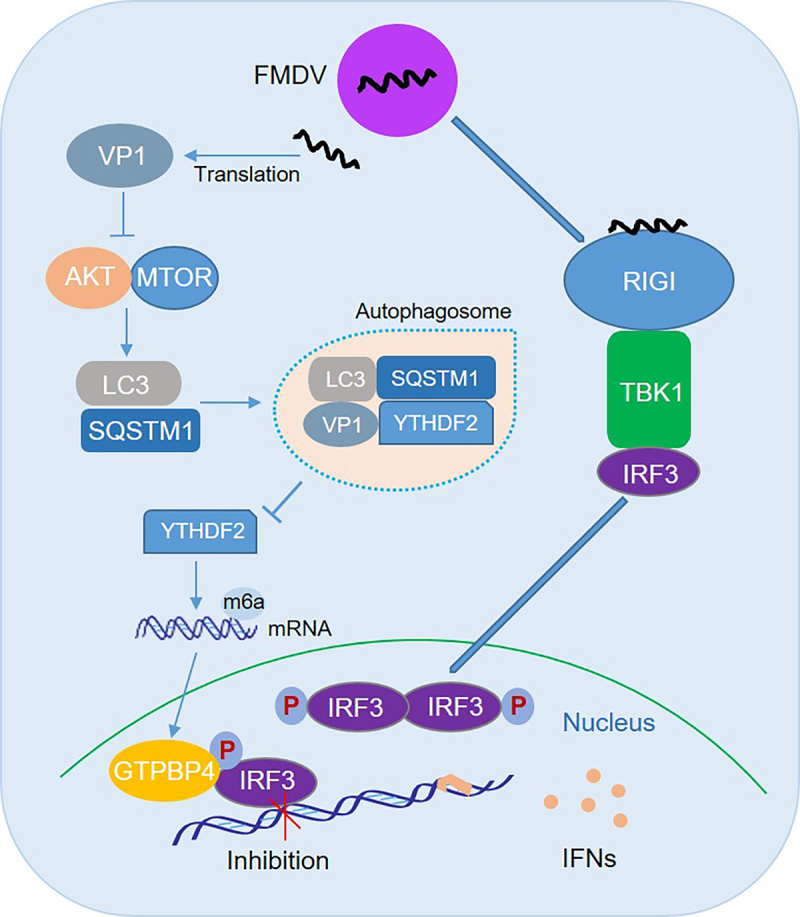
Schematic representation of the model of GTPBP4 and YTHDF2 in innate immune response and autophagy. In this model, FMDV structural protein VP1 interacts with and degrades YTHDF2 in an AKT-MTOR-dependent autophagy pathway, resulting in an increase in *GTPBP4* mRNA and protein levels. Increased GTPBP4 inhibits IRF3 binding to the *IFNB* promoter, suppressing FMDV-induced type I interferon production and promoting viral replication.

Based on our findings, we proposed a model for the role of GTPBP4 and YTHDF2 in antiviral innate immune responses and autophagy (Figure 8). FMDV structural protein VP1 interacts with and degrades YTHDF2 in an AKT-MTOR-dependent autophagy pathway, resulting in an increase in *GTPBP4* mRNA and protein levels. Increased GTPBP4 inhibits IRF3 binding to the *IFNB* promoter, suppressing FMDV-induced type I interferon production and promoting viral replication. In conclusion, our study revealed an underlying mechanism of how VP1 negatively regulates innate immunity through the autophagy pathway, which would contribute to understanding the negative regulation of host innate immune responses and the function of GTPBP4 and YTHDF2 during FMDV infection.

## Materials and methods

### Ethics statement

All animals have handled strictly following good animal practice according to the Animal Ethics Procedures and Guidelines of the People’s Republic of China, and the study was approved by the Animal Ethics Committee of Lanzhou Veterinary Research Institute of the Chinese Academy of Agricultural Sciences (Licence no. SYXK [GAN] 2010–003).

### *Gtpbp4*^*±*^ *mice*

*Gtpbp4/Nog1*^±^ mice (C57BL/6) were purchased from Cyagen Biosciences (S-KO-13001), and maintained in the specific pathogen-free animal facility with free access to food and water. *Gtpbp4*^±^ mice were obtained using the CRISPR-Cas9 system. The single guide RNAs (sgRNAs) targeting exons 2, 3, and 4 were utilized. *Gtpbp4*^±^ mice developed normally and gained weight, similar to WT mice. Mouse experimental work was performed using 3-days-old suckling mice, and was age- and sex-matched in each experiment.

### Detection of FMDV and EV71 in mice

FMDV and EV71 in the mice carcasses without the head, tail, limbs, and viscera were isolated, as described previously [80]. To quantify the number of viral particles, part of the mouse tissue was weighed and homogenized by disposable tissue grinders (VWR 47,732–448) in Dulbecco’s modified Eagle medium (DMEM; Thermo Scientific 11,965,092) supplemented with 1% penicillin-streptomycin-neomycin antibiotic mixture (Thermo Scientific 15,640,055), 2.5 μg/mL fungizone (Thermo Scientific 15,290,026), and 1% L-Glutamax (Thermo Scientific,A2916801). BHK-21 and RD cells are commonly used to determine the titers of FMDV and EV71, respectively [80,81]. Therefore, titration of FMDV and EV71 was performed using BHK-21 and RD cells.

### Immunohistochemical analysis

The immunohistochemical analyses were performed as described previously [82,83]. Briefly, the lung and liver of mice were collected and fixed with 4% neutral formalin (Solarbio, P1110) at room temperature for 3 d. Serial tissue sections were cut into 4-μm thicknesses after embedding in paraffin (Solarbio, YA0012). The available slides were stained with hematoxylin and eosin (Solarbio, G1120). The histological changes were visualized by light microscopy (Olympus, BX41).

### Cells and viruses

PK-15 cells (ATCC, BHC706), HT-29 cells (ATCC, CBP60011), IBRS-2 cells (ECACC 84,100,503), HEK-293T cells (ATCC, CRL-11268), BHK-21 cells (ATCC,

CBP60642), and RD cells (ATCC, CBP60740) were cultured in Dulbecco’s modified Eagle medium supplemented with 10% heat-inactivated fetal bovine serum (Gibco, 10099141C) and maintained at 37°C (5% CO_2_). *ATG7*^−/−^ cells were prepared in our laboratory [84]. FMDV type O strain O/BY/CHA/2010 was used for the viral challenge. The EV71 strain H (VR-1432) stored in our laboratory was used for the viral challenge [71,85]. SeV strain was kindly provided by Prof. Hongbing Shu (Wuhan University, China) and amplified in specific pathogen-free eggs as described previously [63]. The 50% tissue culture infectious dose (TCID_50_) was calculated by using the Reed and Muench method [86].

### Plasmids and antibodies

The cDNAs of *GTPBP4*/*NOG1* and *YTHDF2* were amplified from PK-15 cells and cloned into p3×Flag-CMV-7.1 vector (Sigma-Aldrich, E7533) to yield the N terminal Flag-tagged expression construct (Flag-GTPBP4 and Flag-YTHDF2). The FMDV full-length viral cDNAs were inserted into the p3×Flag-CMV-7.1 vector to construct plasmids expressing Flag-tagged viral proteins. Flag-YTHDF2 and a series of Flag-tagged truncated VP1 were constructed by mutagenesis PCR. HA-IRF3 5D and its mutant expression plasmids were stored by our laboratory previously [87]. The constructed plasmid was analyzed and verified by DNA sequencing. The plasmids were transfected into cells using polyplus-transfection reagent (Jet-PEI 101,000,006), according to the manufacturer′s protocol.

The commercial antibodies used in this study include anti-Flag polyclonal antibody (Sigma-Aldrich, F9291), anti-GTPBP4/NOG1 polyclonal antibody (Abcam, ab92342), anti-MTOR monoclonal antibody (Cell Signaling Technology, 2983), anti-p-MTOR polyclonal antibody (Cell Signaling Technology, 2971), anti-YTHDF2 monoclonal antibody (Cell Signaling Technology 71,283), anti-IRF3 monoclonal antibody (Cell Signaling Technology, 4302), anti-AKT polyclonal antibody (ABclonal, A18120), anti-p-AKT polyclonal antibody (ABclonal, AP1208), anti-ULK1 monoclonal antibody (ABclonal, A8529), anti-p-ULK1 monoclonal antibody (ABclonal, AP0736), anti-SIRT3 monoclonal antibody (ABclonal, A5419), anti-HIF1A monoclonal antibody (ABclonal, A7684), anti-ATG5 polyclonal antibody (ABclonal, A0203), anti-ATG7 polyclonal antibody (ABclonal, A0691), anti-SQSTM1/p62 polyclonal antibody (ABclonal, A19700), anti-YTHDF1 polyclonal antibody (ABclonal, A23773), anti-YTHDF3 polyclonal antibody (ABclonal,A8395), anti-YTHDC2 polyclonal antibody (ABclonal, A15004), anti-LC3 polyclonal antibody (ABclonal, A5618), anti-EV71 3C polyclonal antibody (ABclonal, A23772), anti-m6A monoclonal antibody (Synaptic Systems 202,003), and anti-ACTB/β-actin monoclonal antibody (Sigma-Aldrich, MABT523). Anti-FMDV VP1 polyclonal antibody was prepared in our laboratory [70].

### Coimmunoprecipitation and western blotting

PK-15 cells were mock-infected and infected with FMDV or transfected with various indicated expressing plasmids, and the cells were collected and lysed using RIPA buffer containing protease inhibitors (Solarbio, R0010) at the indicated time points. Afterwards, the cells were immunoprecipitated with the indicated antibodies, as described previously [88].

For western blotting, the cells were collected and solubilized using sodium dodecyl sulfate-polyacrylamide gel electrophoresis (SDS-PAGE) sample loading buffer (Solarbio, P1040). Afterthat, the samples were resolved by SDS-PAGE for western blotting and transferred to an Immobilon-P membrane (Millipore, IPVH00010). The membrane was blocked with 5% skim milk powder (Solarbio, D8340) for 2 h at room temperature, then incubated with primary antibody (1:1000) overnight at 4°C and secondary antibody (1:5000; ABclonal, AS003, AS014) for 1.5 h at room temperature. The antibody-antigen complexes were visualized using westernbright ECL HRP substrate (Thermo Scientific 32,209).

### Knockdown of protein using siRNA

The siRNA in this study was designed and synthesized by Tsingke Biological Technology. Knockdown of endogenous proteins in PK-15 and HT-29 cells was performed by transfecting siRNA. NC siRNA was used as a negative control. According to the manufacturer’s protocol, the siRNA transfection was performed using Lipofectamine 2000 (Thermo Scientific 11,668,019). The porcine *GTPBP4* siRNA sequences are F: GUGUCGAAACCAAGAUGAA, R: UUCAUCUUGGUUUCGACAC. The human *GTPBP4* siRNA sequences are F: GUGUUGACAUGGACGAUAA, R: UUAUCGUCCAUGUCAACAC. The porcine *YTHDF1* siRNA sequences are F: GGCUGGAGAACAACGACAA, R: UUGUCGUUGUUCUCCAGCC. The porcine *YTHDF2* siRNA sequences are F: GGAUCUGGAUCUACUCCUU, R: AAGGAGUAGAUCCAGAUCC. The porcine *YTHDF3* siRNA sequences are F: GGUAAUGCUGAUUUCUCUA, R: UAGAGAAAUCAGCAUUACC. The porcine *YTHDC2* siRNA sequences are F: GAUGCUUAAGACAAUAGAU, R: AUCUAUUGUCUUAAGCAUC.

### RNA extraction and quantitative PCR (qPCR)

Total RNAs in the cells and mice carcasses without the head, tail, limbs, and viscera were extracted by TRIzol reagent (Thermo Scientific 15,596,026). The extracted RNA and HiScript II Q Select RT SuperMix (Vazyme, Q221) were used to synthesize cDNAs. The expression of *IFNB*, *IFNA1*, *ISG15*, *IFIT2*, and *GTPBP4* mRNA was detected using the cDNAs, ChamQ Universal SYBR qPCR Master Mix (Vazyme, Q711), and Mx3005P qPCR System (Agilent Technologies, Mx3005P). The *GAPDH* gene was used as an internal control. The relative expression of mRNA was calculated using the comparative cycle threshold (CT) (2^−ΔΔCT^) method [89]. The qPCR primers sequences are as follows:

porcine *IFNB*-F: GCTAACAAGTGCATCCTCCAAA, R: AGCACATCATAGCTCATGGAAAGA;

porcine *ISG15*-F: GATCGGTGTGCCTGCCTTC, R: CGTTGCTGCGACCCTTGT;

porcine *IFIT2*-F: CTGGCAAAGAGCCCTAAGGA, R: CTCAGAGGGTCAATGGAATTCC;

porcine *IFNA1*-F: CAGGAGGCGGGGCTGGAAGG, R: GAGGGTGAGTCTGTGGAAGT;

porcine *GTPBP4*-F: GCCTTCACCACCAAGTCCCTG, R: CGTCGCATTTGTTCGCTACCA;

porcine *GAPDH*-F: ACATGGCCTCCAAGGAGTAAGA, R: GATCGAGTTGGGGCTGTGACT;

mouse *Gtpbp4*-F: GTCAAATAAATATTGCCAAAAA, R: TACCCACACAAAAGCAGAGTCC;

mouse *Ifnb*-F: GCACTGGGTGGAATGAGACTATTG, R: TTCTGAGGCATCAACTGACAGGTC;

mouse *Isg15*-F: AGTGGTACAGAACTGCAGCGA, R: TGCGTCAGAAAGACCTCATAG;

mouse *Ifit2*-F: TTGACTGTGAGGAGGGGTGGG, R: TGAATTCTGGGTTCTTCGGGT;

mouse *Ifna1*-F: AGGACTCATCTGCTGCTTGGA, R: GGGGCTGTGTTTCTTCTCTCT;

mouse *Gapdh*-F: ACCACAGTCCATGCCATCA, R: TCCACCACCCTGTTGCTGTA.

### Establishment of knockout cell lines using the CRISPR-Cas9 system

The *YTHDF2* and *ATG5* knockout cell line was established, as described previously [90]. The small guide RNAs (sgRNAs) targeting porcine *YTHDF2* and *ATG5* were designed using the online CRISPR design tool (http://crispr.mit.edu/). The sgRNA was inserted into the pLentiCRISPR plasmid with the puromycin selection gene. The constructs were transfected to cells using polyplus-transfection reagent. Cells were selected by puromycin (2.5 μg/mL, Solarbio, P8230) to obtain stable knockout cells. After confirmation of the activity of the designed sgRNA using the T7 Endonuclease I (NEB, E3321), the knockout cell lines were confirmed by western blotting. The sgRNA sequences are as follows. Porcine *YTHDF2*: AATTAAAGCCGGGCCCGAGA.

Porcine *ATG5*: AAGATGTGCTTCGAGATGTGTGG.

### Indirect immunofluorescence assay

Indirect immunofluorescence assay (IFA) was performed as described in our previous studies [87,91]. Briefly, cells cultured on Nunc glass bottom dishes (Thermo Scientific 150,680) were infected with FMDV or transfected with various plasmids. At the indicated time points, the cells were fixed with an acetone-methanol mixture (1:1) for 24 h at 4°C and were blocked and permeated with 5% normal bovine serum (Solarbio, A8010) and 0.2% Triton X-100 (Solarbio, T8200), respectively. Then, cells were incubated with appropriate primary antibodies (1:250) overnight at 4°C and fluorochrome-conjugated secondary antibodies (1:500; Thermo Scientific, A11034) in the dark for 2 h at room temperature. Afterward, the cells were stained with 4’,6-diamidino-2-phenylindole (DAPI; Solarbio, C0060) for 10 min at room temperature to show the nuclei. The fluorescence was visualized using a Nikon Eclipse 80i fluorescence microscope.

### Luciferase reporter assay

HEK-293T cells cultured in 24-well plates were co-transfected with 0.1 μg/well of *IFNB*-Luc along with 0.01 μg/well of pRL-TK Renilla luciferase reporter plasmid and other plasmids. At 24 hpt, the cells were lysed, and the dual-specific luciferase assay kit (Promega Corporation, E1500) was used to analyze the firefly and Renilla luciferase activities, according to the manufacturer′s instructions.

### Chromatin immunoprecipitation (ChIP)

The ChIP assay was performed using a pierce magnetic ChIP kit (Thermo Scientific 26,156), according to the manufacturer′s instructions. Briefly, cells were transfected with various plasmids or infected with FMDV. Then, the cells were harvested and resuspended using RIPA buffer after cross-linking with 1% formaldehyde. The chromatin was sheared into lengths of ∼300 bp by sonication. The lysates were incubated with anti-IRF3 antibody and protein G agarose. “IgG” immunoprecipitation was used as a negative control. The chromatin DNA was then eluted from the beads. Afterward, the bound DNA was extracted using phenol-chloroform and precipitated with ethanol after treatment with proteinase K. The quantity of DNA was determined by qPCR with specific primers. A comparative Ct method was used to assess the relative enrichment of the immunoprecipitated DNA. The abundance of the immunoprecipitated DNA was normalized to the input DNA levels. The primers of the human *IFNB* promoter are as described previously [92]. The primer sequences of the porcine *IFNB* promoter are

F: GGCGGTACCCTTGGCTTATGGTGGTTTTTTTTG,

R: TTTCTCGAGGCTCCACTACTCAAGTGCTGAAG.

### Transmission Electron Microscopy (TEM)

The status of the intracellular autophagy was photographed using a transmission electron microscope, as described previously [78,93]. Briefly, cells were collected and centrifuged at 1000 ×g for 10 min. The pellet was fixed by 3% glutaraldehyde (Sigma-Aldrich, G6257) for 48 h and 1% osmium tetroxide (Sigma-Aldrich, 1.24505) for 1 h and then dehydrated using grades of ethyl alcohol (Sigma-Aldrich, EX0280) and cleared using propylene oxide (Solarbio, YZ1576945). Finally, the cells were embedded in araldite (Sigma-Aldrich 10,951) and polymerized at 60°C for 48 h. The ultrathin sections prepared by Leica EM UC7 ultramicrotome were stained with uranyl acetate (Sigma-Aldrich, CDS021290) and lead citrate (Sigma-Aldrich 15,326). The sections were scanned by JEM 1400 plus TEM at 80 KVA, and images were captured using Gatan SC 1000B camera.

### M6A MeRIP-seq

Total RNA was isolated and purified using TRIzol reagent following the manufacturer’s procedure. Each sample’s RNA amount and purity were quantified using NanoDrop ND-1000, and the RNA integrity was assessed by Bioanalyzer 2100. Poly (A) RNA was purified from 50 μg total RNA using Dynabeads Oligo (dT)_25_ (Thermo Fisher 61,005) by two rounds of purification. Then, the poly(A) RNA was fragmented into small pieces using Magnesium RNA Fragmentation Module (NEB, e6150) at 86°C for 7 min. The cleaved RNA fragments were incubated for 2 h at 4°C with m6A-specific antibody in IP buffer (50 mM Tris-HCl, pH 7.2, 750 mM NaCl and 0.5% Igepal CA-630 [Sigma-Aldrich 18,896]). The IP RNA was reverse-transcribed to cDNA by SuperScript™ II Reverse Transcriptase (Invitrogen 1,896,649). An A-base was added to the blunt ends of each strand, preparing for ligation to the indexed adapters. After the heat-labile UDG enzyme (NEB, m0280) treatment of the U-labeled second-stranded DNAs, the ligated products were amplified with PCR to obtain a sequencing library. Finally, paired-end sequencing was performed using an Illumina Novaseq™ 6000 platform, according to the manufacturer’s protocol.

### RNA-binding protein IP

To prove that YTHDF2 can bind to mRNA of *GTPBP4* directly, PK-15 cells were transfected with Flag-YTHDF2 for RNA IP (RIP) assays, as described previously [42]. Briefly, PK-15 cells transfected with Flag-YTHDF2 were collected and immunoprecipitated using anti-IgG or anti-Flag antibodies. Then, the target gene *GTPBP4* expression was detected by qPCR analysis.

### Methylated m6A RIP-qPCR

Total RNA was isolated and purified using TRIzol reagent following the manufacturer’s procedure. The RNA was incubated at 4°C for 2 h with m6A-specific antibody in IP buffer (50 mM Tris-HCl, pH 7.2, 750 mM NaCl and 0.5% Igepal CA-630). Then, the IP RNA was reverse-transcribed to cDNA. QPCR analysis of the methylated RNA was performed to detect levels of methylated *GTPBP4* mRNA.

### Elisa

The expression of *IFNB* protein in the supernatant and mouse serum was detected using porcine or mouse *IFNB* ELISA kit (Solarbio, SEKP-0046, SEKM-0032), respectively. The measured value was compared with the standard according to the manufacturer′s instructions.

### Statistical analysis

Statistical analysis was performed using SPSS Statistics for Windows, Version 17.0 (SPSS Inc., Chicago, IL, USA). The unpaired *t*-test (two-tailed test analysis) was used in this study. A *P-value <0.05 was considered statistically significant; A **P-value <0.01 was considered statistically significant. Data are presented as mean ± SD.

## Supplementary Material

Supplementary figures R4.docx

Table S1 R3.xlsx
