# Supplementary material for: Foot-and-mouth disease virus VP1 degrades YTHDF2 through autophagy to regulate IRF3 activity for viral replication
Source: Autophagy. 2024 Mar 22;20(7):1597–615. doi: 10.1080/15548627.2024.2330105 (PMC11210904; doi:10.1080/15548627.2024.2330105)
Supplement: Supplementary figures R4.docx [file KAUP_A_2330105_SM1203.docx]

**
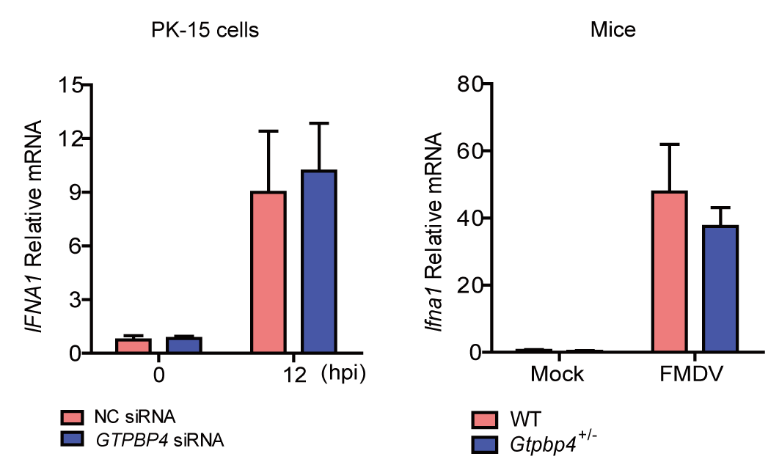
**

**Figure S1.** GTPBP4 did not affect FMDV-induced expression of *IFNA1*. The mRNA expression of *IFNA1* in *GTPBP4* siRNA-transfected cells or FMDV-infected mice was detected by qPCR.

**
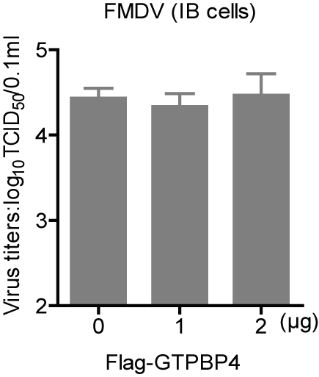
**

**Figure S2.** The impact of GTPBP4 on FMDV replication in IB cells. IB (IBRS-2) cells transfected with increasing Flag-GTPBP4 expression plasmids (0, 1, and 2 μg) were infected with FMDV for 8 h, and the viral titers in the supernatant were measured by TCID_50_ assay.


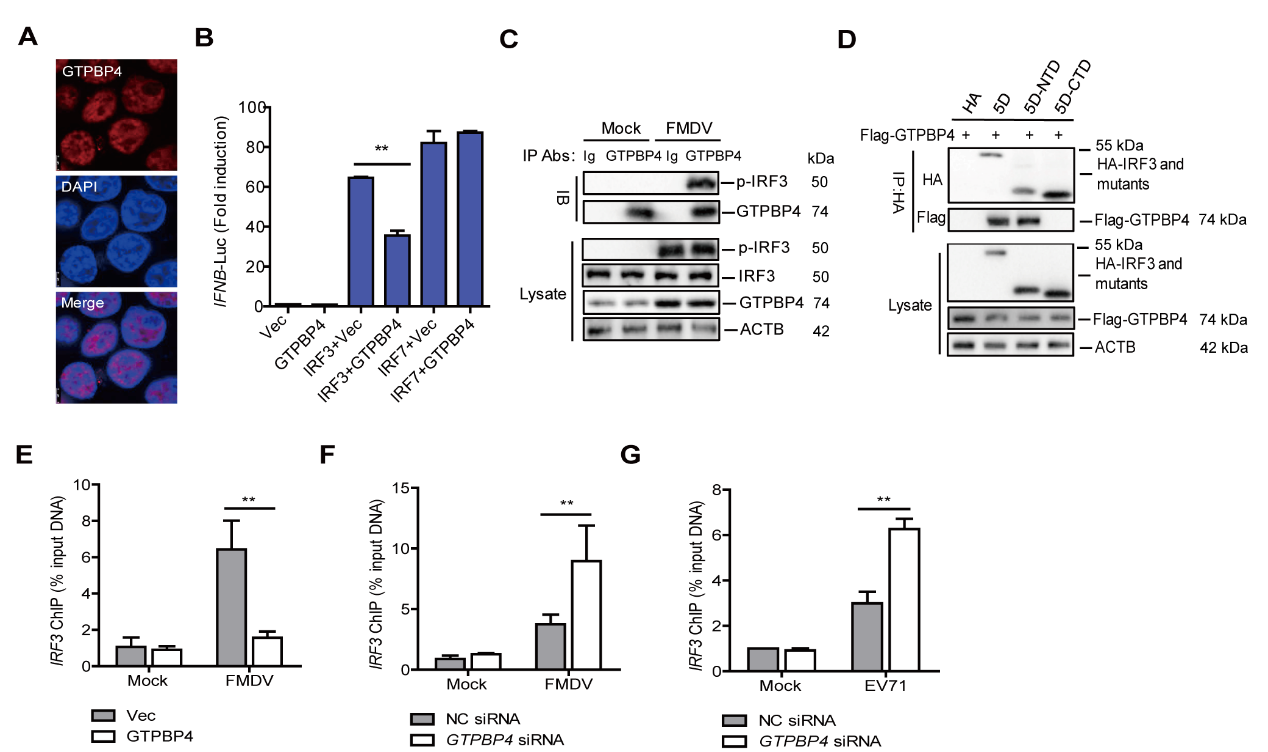


**Figure S3.** GTPBP4 inhibits FMDV-induced interaction between IRF3 and *IFNB* promoter. (**A**) The localization of GTPBP4 was detected by IFA using an anti-GTPBP4 antibody. (**B**) HEK-293T cells were transfected with 0.1 μg/well of *IFNB*-Luc, 0.01 μg/well of pRL-TK plasmid along with 0.1 μg/well of Flag vector or Flag-GTPBP4 expressing plasmid, and HA-tagged IRF3 or IRF7 expressing plasmids. At 24 hpt, the activation of the *IFNB* promoter was detected by the dual-specific luciferase assay kit. (**C**) PK-15 cells were mock-infected or infected with FMDV (MOI 0.1) for 6 h, and the cell lysates were immunoprecipitated with anti-GTPBP4 and anti-IgG antibodies and subjected to western blotting. (**D**) PK-15 cells were transfected with 5 μg of Flag- GTPBP4 expression plasmid along with 5 μg of empty vector, HA-IRF3 5D, or HA-IRF3 5D mutants expression plasmids. The cell lysates were immunoprecipitated with anti-HA antibody and subjected to western blotting. (**E-G**) PK-15 cells transfected with Flag-GTPBP4 expression plasmid (**E**) or *GTPBP4* siRNA or NC siRNA (**F**) were infected with FMDV (MOI 0.1) for 6 h. HT-29 cells transfected with *GTPBP4* or NC siRNA were infected with EV71 (MOI 1) for 12 h (**G**). Chromatin was immunoprecipitated with an anti-IRF3 antibody. The impact of GTPBP4 on IRF3 binding onto *IFNB* promoter was analyzed by quantitative ChIP assay.


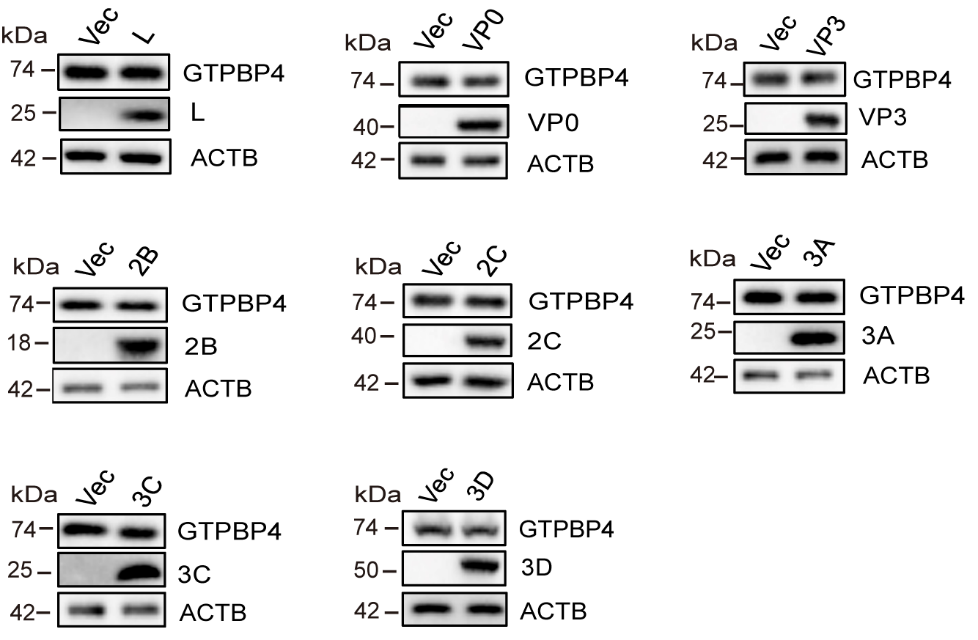


**Figure S4.** The impact of viral proteins on GTPBP4 expression. PK-15 cells were transfected with 2 μg of plasmids expressing Flag-tagged viral proteins. The protein expression of GTPBP4 was detected by western blotting.


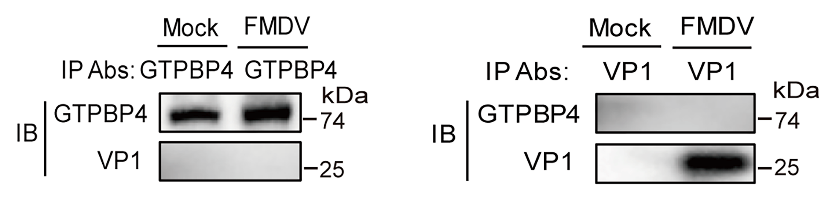


**Figure S5.** GTPBP4 did not interact with FMDV VP1. PK-15 cells were mock-infected or infected with FMDV (MOI 0.1) for 8 h. The cell lysates were immunoprecipitated with anti-GTPBP4 (left) or anti-VP1 (right) antibodies. The antibody-antigen complexes were analyzed by the indicated antibodies.


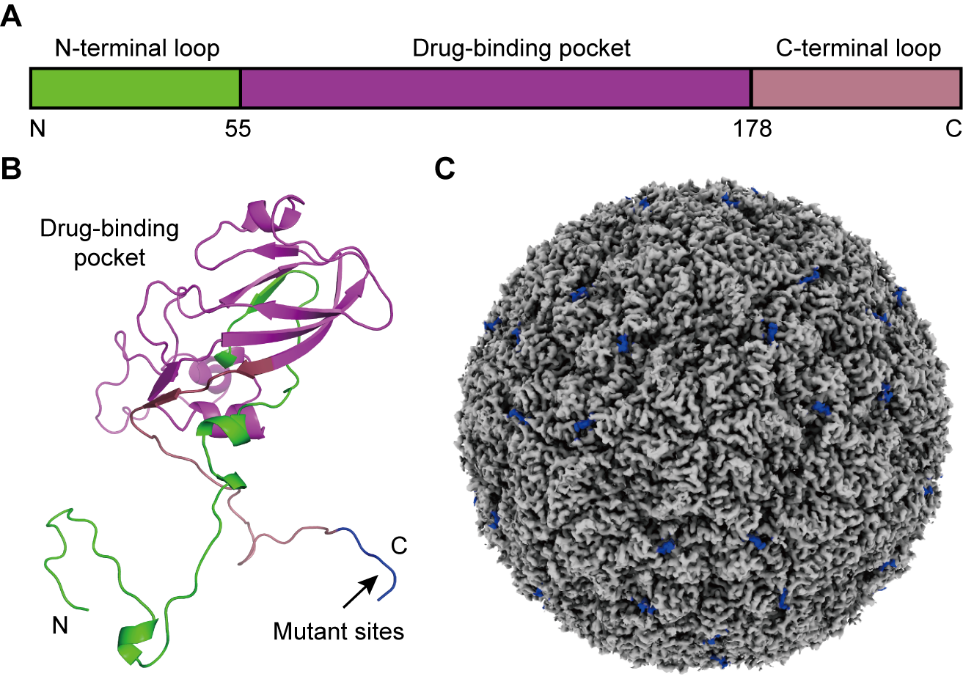


**Figure S6.** Schematic representation of the structure and functional regions in FMDV VP1. (**A-B**) The structure and functional regions of VP1. (**C**) The 209th site of VP1 (blue) is mainly distributed on the surface of FMDV.


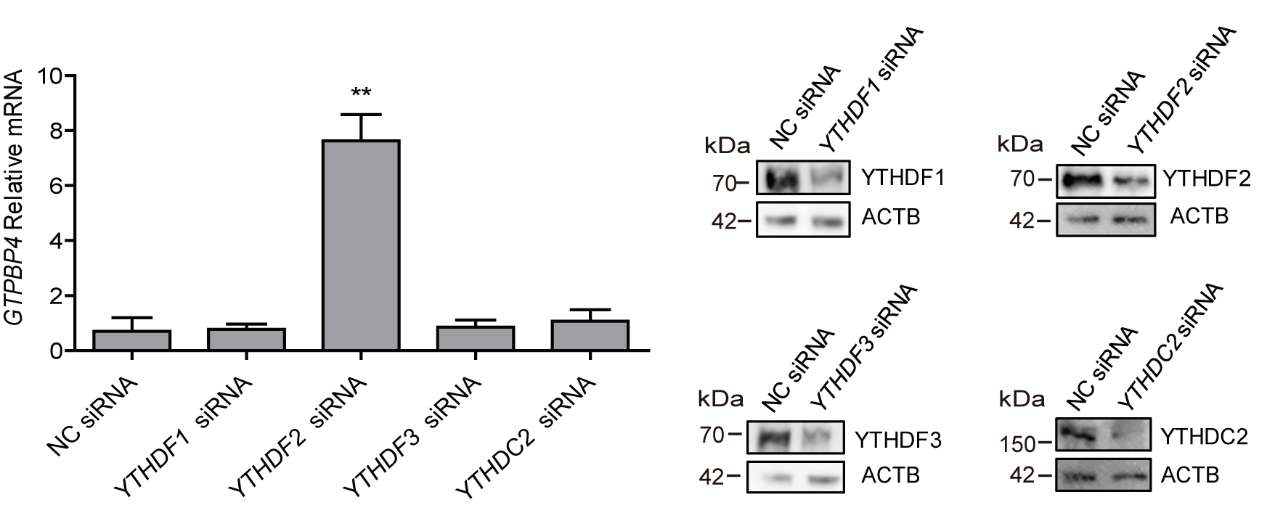


**Figure S7.** YTHDF2 was associated with *GTPBP4* mRNA expression. PK-15 cells were transfected with 150 nM of *YTHDF1*, *YTHDF2*, *YTHDF3*, *YTHDC2* siRNA or NC siRNA. The expression of the indicated proteins and *GTPBP4* mRNA was detected by western blotting and qPCR, respectively.


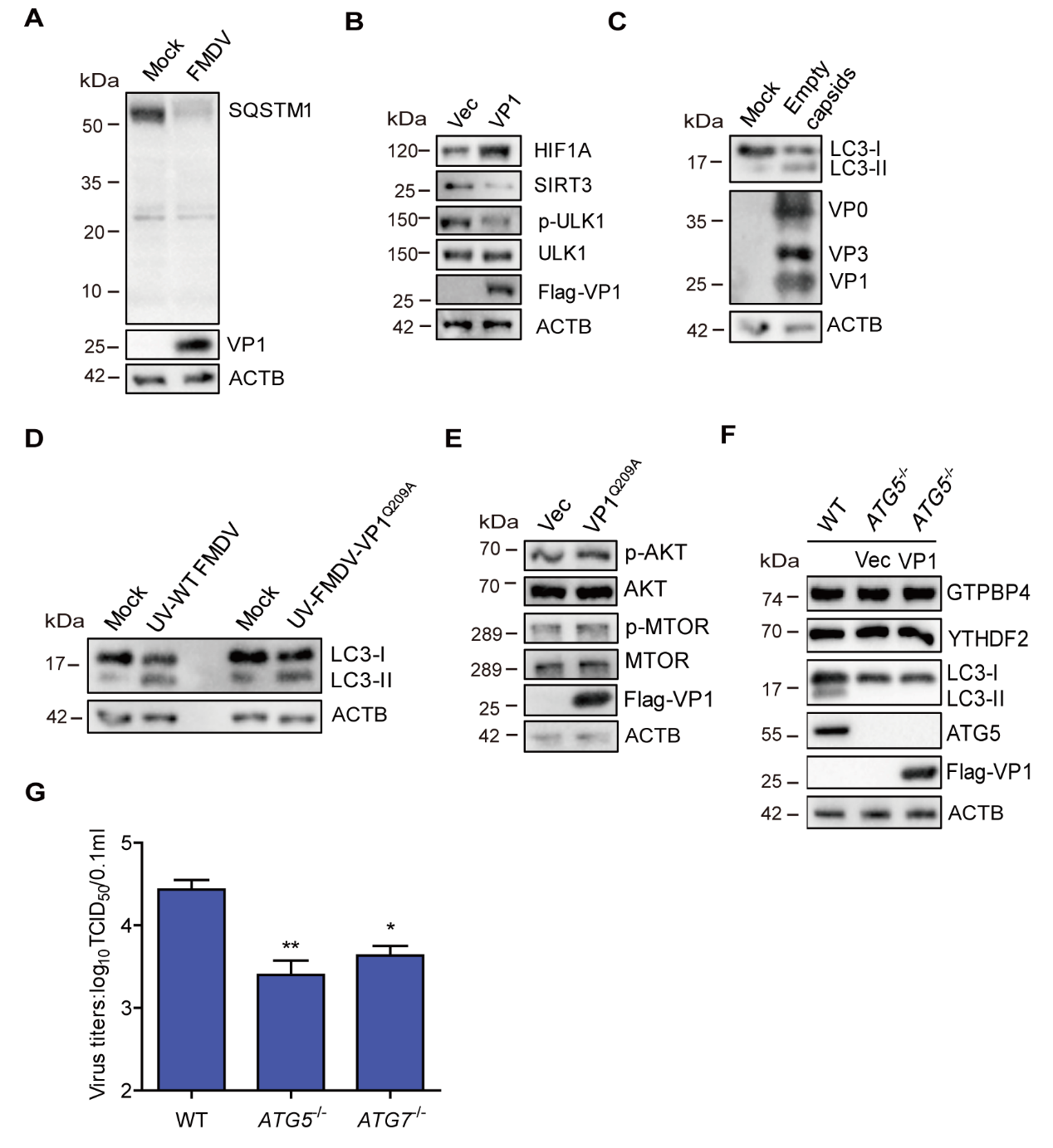


**Figure S8.** The impact of VP1 on autophagy. (**A**) PK-15 cells were mock-infected or infected with FMDV, and the expression of SQSTM1 protein was detected by western blotting. (**B**) PK-15 cells were transfected with 2 μg of empty vector or Flag-VP1-expressing plasmids for 24 h. The cells were collected and subjected to western blotting analysis. (**C**) PK-15 cells were incubated with empty capsids of FMDV (200 μg/mL). The cells were collected and subjected to western blotting analysis. (**D**) PK-15 cells were mock infected or infected with UV inactivation of WT FMDV or FMDV-VP1^Q209A^ for 4 h (MOI 10). The cells were collected and subjected to western blotting analysis. (**E**) PK-15 cells were transfected with 2 μg of empty vector or Flag-VP1^Q209A^-expressing plasmids. The expression of MTOR, p-MTOR, AKT, p-AKT was detected by western blotting. (**F**) *ATG5*^-/-^ cells were transfected with 2 μg of empty vector or Flag-VP1-expressing plasmids for 24 h. The expression of GTPBP4, YTHDF2, LC3, and ATG5 was detected by western blotting. (**G**) WT, *ATG5*^-/-^, and *ATG7*^-/-^ cells were infected with FMDV, and the viral titers were determined by TCID_50_ assay.
